# A systematic review of analytical methods used in genetic association analysis of the X-chromosome

**DOI:** 10.1093/bib/bbac287

**Published:** 2022-07-29

**Authors:** Nick Keur, Isis Ricaño-Ponce, Vinod Kumar, Vasiliki Matzaraki

**Affiliations:** Department of Internal Medicine and Radboud Center for Infectious Diseases, Radboud University Medical Center, Geert Grooteplein Zuid 10, 6525 HP, Nijmegen, The Netherlands; Department of Internal Medicine and Radboud Center for Infectious Diseases, Radboud University Medical Center, Geert Grooteplein Zuid 10, 6525 HP, Nijmegen, The Netherlands; Department of Internal Medicine and Radboud Center for Infectious Diseases, Radboud University Medical Center, Geert Grooteplein Zuid 10, 6525 HP, Nijmegen, The Netherlands; Department of Genetics, University of Groningen, University Medical Center Groningen, Hanzeplein 1, 9700RB, Groningen, The Netherlands; Department of Internal Medicine and Radboud Center for Infectious Diseases, Radboud University Medical Center, Geert Grooteplein Zuid 10, 6525 HP, Nijmegen, The Netherlands

**Keywords:** X-chromosome, GWAS, eQTL, X-inactivation, association analysis, linear regression

## Abstract

Genetic association studies have been very successful at elucidating the genetic background of many complex diseases/traits. However, the X-chromosome is often neglected in these studies because of technical difficulties and the fact that most tools only utilize genetic data from autosomes. In this review, we aim to provide an overview of different practical approaches that are followed to incorporate the X-chromosome in association analysis, such as Genome-Wide Association Studies and Expression Quantitative Trait Loci Analysis. In general, the choice of which test statistics is most appropriate will depend on three main criteria: (1) the underlying X-inactivation model, (2) if Hardy–Weinberg equilibrium holds and sex-specific allele frequencies are expected and (3) whether adjustment for confounding variables is required. All in all, it is recommended that a combination of different association tests should be used for the analysis of X-chromosome.

## Introduction

Genome-wide association studies (GWAS) are often considered the first-choice method for identification of associations between genetic variation and complex phenotypes. Generally, these studies are performed in a case/control setting and aim to identify genetic variants associated with a disease or other complex phenotypes of interest. Over the last decades, tens of thousands of genetic variants have been identified by such studies which affect and/or influence the risk for complex diseases/traits [1]. However, the X-chromosome is often neglected in genetic association studies, and, as a consequence, the X-chromosome is severely underrepresented in most published association studies to date [1]. Many studies have shown suggestive statistical evidence that a great number of complex diseases/traits are sexually dimorphic (differ between males and females), which points toward a potential contribution of the X-chromosome [2, 3]. The human X-chromosome is 155 Mb and has over 800 protein-coding and 600 non-coding genes, and many are known to be involved in the regulation of the human immune system [4]. In addition, it is estimated that approximately 10% of all microRNAs (miRNA) found in human cells are located on the X-chromosome. While the role of most miRNAs is unclear, several are known to have important functions in immunity [5, 6].

There are several reasons which make analysis of the X-chromosome more complex compared with autosomes. One main reason is its unique genetic make-up, with female cells carrying two copies of the X-chromosome [7], while males only have one copy of the X-chromosome. For example, as males carry only one allele, the signal intensities obtained from standard genotyping arrays are lower than for females who carry two alleles. This needs to be adequately addressed in the genotype-calling step and has further consequences for genotype imputation. In addition, this leads to reduced sample size in allelic variants and therefore greatly affects the statistical power to detect associations. To compensate for imbalances in gene dosages in female cells, the X-chromosome undergoes a process called X-chromosome inactivation (XCI) [7]. This is of importance when deciding how to test for association with X-chromosomal variants as discussed next.

In the early stages of embryo-genesis in female cells, one version of the X-chromosome is randomly epigenetically silenced. As a result, female cells become cellular mosaic, which means that approximately half of cells express maternal genes, while the rest expresses paternal genes [2]. This biological process is complex and is known to vary extensively between individuals with regard to which genetic regions will be silenced within and across different tissues [8, 9]. In addition, this process has been shown to be impaired upon aging [10]. However, some genetic regions are known to escape this process, such as the pseudo-autosomal region (PAR), so it is normally expressed from both X-chromosomes in females. The PAR region is located at the start and end of the X-chromosome, and helps pair and segregate the chromosomes during meiosis. While many genes located on the X-chromosome undergo some form of XCI, there are genes known to fully and/or partially escape silencing. A small fraction of the heterogeneity observed among females in X-linked genes has been reported to fully escape XCI (around 15%) while others exhibit variable patterns of XCI (around 10%), which potentially contributes to the disparity in disease risk and pathogenesis [11].

In addition, X-chromosome is subject to lower mutation rates compared with autosomes [12], which results in reduced genetic diversity as well as smaller interspecies divergence [13]. Due to its unique properties, X-linked genes have distinctive inheritance patterns compared with autosomal ones, making it of critical importance to study the genetic architecture of X-linked genetic disorders. To account for these unique properties, several specific association tests have been developed to overcome the challenges when analyzing the X-chromosome [14, 15]. X-chromosome analyses need to be adequately addressed during association analysis, which requires special attention during pre-processing steps, such as quality control and imputation of genetic data [15, 16, 17]. In this review, we aim to provide an overview of current best practices and statistical approaches on how to study genetic effects on the X-chromosome using GWAS and Expression Quantitative Trait Loci Analysis (eQTL) mapping. Finally, we will summarize the advantages and limitations of these approaches for certain diseases [18].

## Advances in computational approaches for X-chromosomal genotype data

### Chromosome-X imputation

In the past, genotyping arrays contained relatively few markers on the X-chromosome, especially when compared with autosomes, resulting in poor representation of X-chromosomal genetic variation. Although the number of markers on the X-chromosome has substantially increased in recent years, the difference in coverage of single nucleotide polymorphisms (SNPs) on different genotyping platforms is still important to consider when performing genetic association studies [19]. Due to generally lower coverage of markers on the X-chromosome, which is especially true for the oldest generation of genotyping arrays, imputation is of critical importance for genetic analysis. Imputation allows filling missing variants that otherwise would not be available, which is necessary when combining genetic data from multiple studies, such as in meta-analysis. Given the unique genetic makeup of the X-chromosome, imputation of variants located on the X-chromosome requires special processing. An often followed approach is to first separate the genetic data from females and males and perform the imputation separately. Since female cells have two copies of the X-chromosome (diploid), imputation is not different than in autosomes and requires no additional steps or special data handling.

However, special attention needs to be taken when imputing SNPs in the PAR region as it has been previously reported that the quality of imputation is worse compared with autosomes, rendering mostly too few SNPs to be considered for analysis [15]. To account for the PAR region, the genetic data from males need to be split based on the human pseudo-autosomal region (PAR) into PAR (diploid) and non-PAR (haploid) regions. Several imputation tools have established dedicated workflows for the imputation of variants on the X-chromosome. For instance, tools that have been used for imputation, such as IMPUTEv2 and higher versions [20], have specific options that enable the imputation of variants of the X-chromosome. Other tools, such as the web-based Michigan imputation server (Minimac) [21], automatically handles the imputation of the X-chromosome. Particularly, the X-chromosomal data are split into three independent chunks (PAR1, non-PAR, PAR2) and are processed separately. These chunks are then automatically merged and returned as one complete X-chromosome file for further downstream analysis.

### Quality control

Following imputation, careful quality control (QC) of genetic data is required before carrying out the association analysis. Although QC is study specific and depends on several factors, such as sample size, imputation accuracy, among others, standard thresholds have been established (Table 1; based on the work of Ziegler et al. [22]). Generally, quality checks are performed on the individual/sample level, which includes removal of individuals with low call rates and excess heterozygosity, with the latter being suggestive of low sample quality or potentially inbreeding. Subsequently, checks are performed for each marker, which includes but are not limited to missingness, minor allele frequency (MAF) and removal of markers that deviate from Hardy–Weinberg equilibrium (HWE). Mixed-sex data often require additional checks with regard to differences between sex. In particular, no significant differences in call rate and heterozygosity should be observed between the sexes and/or between cases and controls in case of a case/control study design [23].

**Table 1:** Overview of quality control filters utilized for genetic data

Sample filters	Threshold
Call-rate	>95%
Cryptic relatedness	Study specific
Population structure	Study specific
Excess heterozygosity	Within mean $\pm $ 3 SD
SNP filter:	
Minor allele frequency	>1%
Missing frequency	<2%
Hardy–Weinberg equilibrium	*P* <10$^{-6}$
Sex-specific filters:	
Missingness by sex	<2%
Heterozygotes by sex	Within mean $\pm $ 3 SD
Call rate between sexes ^*^^*^	>95%
X-chromosome specific filters:	
Proportion of heterozygotes in males ^*^^*^	
Missingness by sex ^*^^*^	

Note: ^*^^*^ specific to the X-chromosome

In addition to the standard quality checks described above, inclusion of X-chromosomal data requires additional checks that are specific to the X-chromosome [15, 24, 25]. First, the inclusion of X-chromosomal data allows the comparison between sex information based on genotype and phenotypic sex data. Individuals with divergent sex information should be excluded from further follow-up analysis. Other X-specific filters include the removal of SNPs, which significantly differ in MAF between males and females, or between cases and controls as these could cause inflated type 1 errors. Last but not the least, whether variants are within HWE needs to be considered as discussed in the next paragraph. Several tools can be used to carry out QC on genetic data, such as PLINK [26], GWAStools [27]

## Test statistics for associations on X-chromosome

Associations between disease and genetic markers are often identified using case/control study designs where the frequency distributions of genotypes are directly compared between cases and controls. However, methods used for autosomal genotype data cannot directly be used for the analysis of X-chromosome because they can potentially lead to statistical inaccuracies. This is mainly due to the need to account for the genetic imbalance between females and males and expected XCI model [28, 29, 30].

To overcome these X-specific challenges, several statistical tests have been proposed in literature, which are specific for the genetic analysis of X-chromosome. These tests make different biological assumptions. For instance, tests can significantly differ with regard to whether to account for XCI, underlying genetic models, and whether HWE or equal allele frequencies in both sexes are assumed. Another consideration that must be taken into account when analyzing the X-chromosome is that the underlying XCI state is not always known. This subsequently can lead to account for X-linked genomic regions that could lead to false positives. Therefore, tools have been developed that can be utilized to make an informed decision regarding the most appropriate XCI model to use for association testing. One such tool is GCTA [31], a genetic toolset that can be used to estimate the total percentage of variance explained by SNPs under different assumptions regarding dosage compensation for the X-chromosome. Hypothesis testing can be performed by comparing the likelihood of each model fitting under the three assumptions (no dosage compensation, full dosage compensation, equal genetic variance). Another tool used to estimate the degree of XCI is XCIR [32], which can be used to identify genes that escape XCI using bulk RNA-seq data and exploits allele-specific expression (ASE). In the next paragraph, we will discuss a number of existing association tests and the underlying assumptions for the analysis of X-chromosome. For more details and further information regarding test statistics selection, we suggest the following previously published studies [14, 16, 28, 29, 33].

### Assumption 1: Escape X-inactivation

One approach to analyze the X-chromosome under the assumption of escape XCI is proposed by Zheng et al [29]. In this approach, several different association test statistics are proposed that each handles the particularities associated with the X-chromosome. Each test performs best under different circumstances and makes different assumptions regarding HWE, underlying genetic model, and sex-specific allele frequencies (Table 2, adapted from [15]). Importantly, these tests do not directly account for the effects of XCI and therefore indirectly assume escaping of the XCI process for the full X-chromosome. The first two proposed test statistics Z_A_ and Z_mfA_ are based on the commonly used allele-based test and require variants to be in HWE. The Z_C_ and Z_mfG_ are genotype-based and are robust to departures from HWE. While the allele-based tests are based on differences in MAF, the genotype-trend test directly compares the genotype distributions between cases and controls [29]. In addition, the tests assume that both females and males have the same risk alleles. For this reason, the latter two test statistics are only applicable when females and males have different risk alleles. In practice, HWE can only be estimated for females as we usually do not know whether HWE holds in males. Therefore, it is suggested by the authors to use a combination of test statistics and rank variants to make informed decisions and select the optimal test statistics [29]. Generally, Z_A_ and Z_mfA_ have good performance under different genetic models given that HWE holds and escape XCI is assumed [29, 34]. Lastly, tools such as Plink [26] or FM_01_ from XWAS [14] can be utilized as a regression-based approach under the assumption of escape XCI. Both tools fit a logistic regression model, whereby default sex is added as a covariate to account for sex-specific allele frequencies. By default, genotypes for males are encoded as 0 / 1 and females as 0 / 1 / 2, and assume equal effect size between males and females and escape XCI.

**Table 2:** Overview of association tests used for the analysis of the X-chromosome

Name	Model	XCI	Sex-stratified	Assumptions	Tools
Z^2^_A_	Allele-based	Escape	No	HWE, Equal alleles	
Z^2^_C_	Genotype-based	Escape	Yes		
Z^2^_mfA_	Allele-based	Escape	Yes	HWE	
Z^2^_mfG_	Genotype-based	Escape	Yes		
T_A_	Additive	Random	No	Equal alleles	snpStats
T_AD_	Additive/Dominant	Random	No	Equal alleles	snpStats
FM_01_^*^^*^	Additive	Escape	Yes		Plink, XWAS
FM_02_^*^^*^	Additive	Random	Yes		Plink, XWAS
FM_F_	Additive	Escape/Random	Yes		XWAS
FM_S_	Additive	Escape/Random	Yes		XWAS
MAX_LR_^*^^*^	Additive	Skewed	No		SkewXCI
XCMAX4^*^^*^	Additive	Skewed	No		XCMAX4

Description of each column in the table. Name: Name of test statistic; Model: Genetic model used by each method; XCI: XCI status assumed by each test; Sex-stratified: Whether the test is performed stratified by sex; Assumptions: Required assumptions for each method; Tools: Provides a tool with an implementation of each method. Note: ^*^^*^ Inclusion of sex as an covariate make these approaches robust to sex-specific allele frequencies

### Assumption 2: Random X-inactivation

An alternative X-specific version of a common autosomal statistical test, which accounts for random XCI, has been proposed by Clayton et al. [35]. For this, Clayton et al. proposed two test statistics. The first test is T_A_, which is similar to the Cochran–Armitage trend test and combines male and female genotypes. These tests do not suffer from reduced power, which could occur when stratifying by sex. In addition, it is robust to departures from HWE given that genotypes are directly compared. Whereas T_A_ is suitable in situations where an additive model is assumed, T_AD_ can be applied when the underlying genetic model is either additive or dominant. This approach was shown to remain valid even when the phenotype between females and males varies, provided that allele frequency does not differ between the two sexes [34, 36]. In general, these tests are equivalent to a Pearson’s Chi2 test in which genotype data from males and females are combined into one combined contingency table. Clayton’s approach has been implemented as an R package in SnpStats [37]. Alternatively, if adjustment for confounding is necessary, tools such as SNPtest, or the FM_02_ from XWAS [14] can be utilized to perform regression-based association testing. Both tools fit logistic regression models and assume random XCI in females and require equal effect size between males and females. By default, sex is added as a covariate and is therefore robust under sex-specific allele frequencies. Genotypes for males in SNPtest are encoded as 0 / 1 and females as 0 / $\frac{1}{2}$ / 1, while in Plink males are encoded as 0 / 2 and females as 0 / 1 / 2. In both cases, the genotype enters as a linear term and the heterozygous genotype falls midway between the homozygous genotypes on the linear predictor scale [33, 37]. This encoding is appropriate because females with heterozygote genotypes have approximately half of the cells active with the major allele, while the other half have the major allele inactive due to random XCI.

### Assumption 3: Skewed or partial X-inactivation

All above-mentioned statistical tests either account explicitly for full escape from XCI or assume random XCI. However, studies have shown that deviations, such as skewed or non-random XCI, are a biological plausibility. [9, 38]. Due to the complexity of XCI and the heterogeneity between genetic regions, the true underlying XCI state is generally unknown. [9, 38]. For this reason, Wang et al [33] proposed a novel approach which utilizes a maximizing likelihood ratio test and considers skewed XCI as an additional possible XCI state. This approach was compared with both Zheng’s and Clayton’s approaches to show that it achieves increased power when the underlying XCI model is non-random or skewed, while it loses some power when XCI is random or escape. In this approach, the three possible genotypes are coded in females as 0, $\gamma $ and 2, where $\gamma $ is an unknown parameter. This $\gamma $ parameter takes possible values between 0 and 2 and can be utilized to quantify the degree of skewness. However, because *P*-values are estimated using a permutation-based approach, which is computationally demanding and time consuming, especially when utilized in GWAS. This approach was further optimized and extended in a later publication [30] to estimate the degree of skewness and make a selection regarding the underlying XCI pattern for each tested marker.

More recently, a novel robust genetic association test has been published by Su et al [39]. In this method, the authors take a similar approach to Wang et al, in which random or escape XCI, but also deviations, such as those observed in skewed XCI, can be assumed. Such deviations are defined as >75% or <25% active alleles in each state. This method was implemented as a regression-based approach, which allows for computationally adjusting for any confounding effects, such as in situations where sex-specific allele frequencies are observed between cases and controls. Unlike the method proposed by Wang et al, which uses a permutation-based approach to estimate *P*-values, this method analytically calculates *P*-values, which makes it more computationally efficient, especially when the full chromosome is scanned for associations as in GWAS. This method was directly compared with others proposed in the XWAS tools, such as FM_01_, FM_02_, FM_S_ and FM_F_ [14]). It has been shown to maintain a good statistical power over a variety of scenarios, especially when the underlying XCI model deviates from random or skewed XCI [39]. This method is implemented using an R script (SkewXCI), which can be found in Table 3.

**Table 3:** Overview of available tools used for the analysis of X-chromosome

Name	Method	Platform	Weblink
Impute2	Imputation	Unix/Win	Link
PLINK	Quality control	Unix/Win	Link
GWASTools	Quality control	R package	Link
SNPTEST	Association testing	Unix/Win	Link
snpStats	Association testing	Unix	Link
XCMAX4	Association testing	R package	Link
XCIR	XCI Inference	R Package	Link
SkewXCI	XCI Inference	R Package	Link
XWAS	Pipeline/Workflow	Unix/Win	Link
GCTA	Association testing	Unix/Win	Link
	XCI Inference		
Matrix eQTL	Association testing	R package	Link

### Sex-stratified analysis

Another approach to test for associations between a disease/trait and genetic markers on the X-chromosome is by analyzing the genetic data in a sex-stratified manner. This approach can be particularly relevant for the analysis of X-chromosome since variants located on this chromosome are more likely to have sex-specific effects regarding disease risk [40, 41, 42]. When the effect is only observed in one sex, or differs between females and males, a sex-stratified association test can identify significant associations. However, one of the downsides is that data stratification based on sex results in a reduction in sample size and, subsequently, reduces statistical power to detect associations [43]. This approach analyzes both males and females separately, and results are thereafter combined to obtain a sex-stratified significance level. This approach has been implemented by Goa et al. in the XWAS framework [14] using Fisher’s (FM_F_) or Stouffer’s (FM_S_) method. Whereas FM_F_ allows SNPs to have different and/or opposite effects on disease risk in males versus females, FM_S_ accounts for potential differences by weighting sample sizes in cases and controls. Both approaches are implemented using logistic regression assuming an additive model. In addition, this approach is insensitive to genotype encoding as the X-chromosome is being analyzed separately in males and females. Lastly, one makes no assumptions with regard to XCI.

## X-chromosome quantitative trait locus analysis

A commonly used approach to follow up on GWAS findings is by performing eQTL mapping. This approach aims to identify target genes whose function or expression is directly influenced by the GWAS-associated variants or by highly correlated variants in linkage disequilibrium (LD) [44]. Identifying genes, which are affected by variants in cis (local) or trans (distant), will help elucidate the underlying biological pathways that influence the observed/studied trait variation or disease risk. An often-used method for eQTL mapping is linear regression, which is considered the most computationally efficient and allows for adjustments using covariates. Although numerous studies have investigated the associations between polymorphisms and gene expression at the genome-wide level, only few studies have included the X-chromosome in their analysis. Next, we will discuss a previously described approach to investigate the impact of sex and genetic variation on gene expression levels, in which X-chromosome was included [40, 41].

### Sex-informed approach

Kukurba et al. followed two approaches, a joint and a sex-stratified analysis, to test whether genetic variants on autosomes and X-chromosome influence gene expression. For this, they used genetic and gene expression data from 922 individuals of European ancestry [41]. To account for XCI, they used the encoding 0, 1, 2 for female genotype data and 0, 2 for male genotype data [41]. This encoding computationally accounts for the hemizygous state in males and assumes random XCI in females. In addition, they used random sampling to ensure that differences in sample sizes did not impact observed differences in gene expression. They first performed a joint analysis, where they combined the data for males and females, to identify cis-eQTLs within a 100 kb window using a linear model with sex as a covariate (Equation 1).The joint analysis was restricted to only genes expressed in both sexes and showed a significant depletion of cis-eQTLs on the X-chromosome compared with the autosomes. Specifically, they observed cis-eQTLs in 74.8% of autosomal genes, while this was only in 43.7% of genes located on the X-chromosome.

(1) \begin{align*} \gamma=\beta1 * genotype + \beta2 * sex + \epsilon \end{align*}

To exclude the possibility that this observation was either a result of male hemizygosity or influenced by XCI in females, they performed an eQTL analysis in a sex-stratified manner. Although the sex-stratified analysis suffers from power loss due to reduced sample size, it can potentially identify weakly associated eQTLs in one sex, whose effect is likely to be diluted when both sexes are analyzed simultaneously [40]. However, depletion of eQTLs was also observed in the sex-stratified analysis, with a pronounced decrease in the number of genes with eQTL effect in males compared with females. This might indicate that the reduced genetic diversity on the X-chromosome may potentially result in the depletion of eQTLs on this chromosome [41, 45]. In addition, lower effect sizes of X-chromosome-specific eQTLs were observed compared with autosomes, which potentially makes the detection of eQTLs more difficult on the X-chromosome compared with autosomes.

(2) \begin{align*} \gamma=\beta1 * genotype + \beta2 * sex + \beta3 * (genotype * sex) + \epsilon \end{align*}

Furthermore, they aimed to identify genetic variants that interact with sex (sex-interacting eQTLs). To detect eQTLs that have some interaction with sex, they applied a linear model with an interaction term for genotype-sex (Equation 2). These interactions usually occur when, for instance, (1) the effect of genotype on expression differs between sexes or (2) when effects may be present in only one of the two sexes or (3) when effects potentially have different magnitudes or opposing directions between the two sexes [46]. Of note, a significant enrichment for sex-interacting eQTLs was observed on the X-chromosome relative to the autosomes, which was in concordance with other studies [40]. In addition, they tested whether the identified sex-interacting eQTLs were enriched for differentially expressed (DE) between the two sexes. They found no enrichment of DE genes with sex-interacting eQTLs, which suggests that these eQTLs are not a direct consequence of differences in gene expression between the sexes. The observed absence of enrichment can be due to other biological factors that differ in a sex-specific matter, such as differences in transcription factor activity, hormone receptors and chromatin accessibility [40, 46].

Last but not the least, in a recently published study [47], the authors performed a sex-biased eQTL (sb-eQTL) analysis on 491 694 conditionally independent cis-eQTLs identified in the sex-combined cis-eQTL analysis using the data from the GTEx v8 project. The linear model described in Equation 2 above was used for the analysis. A total of 369 sb-eQTLs corresponding to 366 genes were identified (FDR $\leq $ 0.25). Interestingly, the sb-eQTLs seem to be more tissue specific than combined eQTLs. For 58% of the sb-eQTLs, both males and females have the same direction of the effect, but different effect sizes. The authors hypothesized that the sb-eQTLs analysis might be affected by the variation on cell type composition that is affected in a sex-specific manner.

## Tools for the genetic analysis of X-chromosome

Although specific tools for analysis of the X-chromosome have been developed in recent years, they are still significantly underutilized in most research [15, 17]. For this reason, we have listed several tools for the genetic analysis of X-chromosome and indicate which ones have options specific to the X-chromosome (Table 3).

## X-chromosome in GWAS: Where are we?

Although the amount and quality of available methods to include the X-chromosome has improved over the years, the number of associations reported is still smaller compared with the ones in autosomes. For instance, a recent review reported that only 437 associations to 268 diseases were reported in the GWAS catalog up until November 2020 [3]. Some of the reasons that might have prevented detection of significant associations on this chromosome could be variations in power, sample size and genotyping arrays across GWA studies [17]. However, probably the main reason is that the X-chromosome has been excluded from most of the GWA studies performed over the past years. Given that the X-chromosome contains 5% of the genes in the human genome [17], many interesting biological insights could be revealed if we include the X-chromosome in future GWASs.

There were multiple reasons why researchers were excluding the X-chromosome in their studies. For example, to name a few, difficulty to generate the genotypes, the complexity in the genetic makeup of the chromosome and the lack of specific guidelines for the analysis. One of the limitations we have addressed in this review is the reduced SNP coverage in the X-chromosome in the commercial genotyping SNP arrays. However, SNP coverage of the X-chromosome has improved tremendously in many of today’s GWAS arrays. Moreover, modern sequencing technologies have also improved the covering of the X-chromosomal regions. Although available genetic data in which the X-chromosome was included might not be perfect, the analysis of such existing underutilized data could enhance discovery and further understanding of the genetics of human disease [11, 17]. Another limitation in X-chromosome analysis is that we now represent the sex chromosome as a diploid in the assembly of X-chromosome. While this assumption holds for the non-recombinant and divergent regions, the homologous PAR regions and the end of the X and Y chromosome are represented twice. If not controlled properly in the analysis, this might cause problems for the interpretation of short-read sequencing [48]. Although this might represent some bias for the human genome, future genome references should consider this.

**Figure 1:**
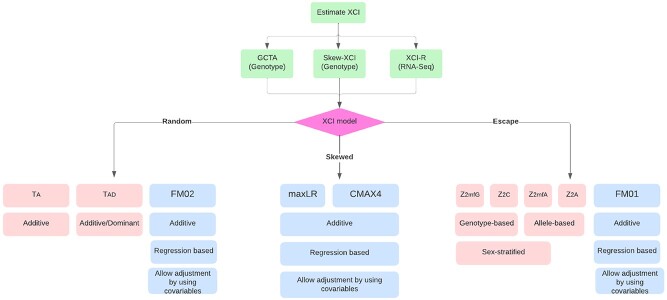
Flowchart of the criteria for selecting the most appropriate test statistics for X-chromosome analysis. The green boxes present tools that can be used to help make a selection regarding XCI (XCI inference), and the blue and pink colored boxes correspond to test statistics and regression-based approaches, respectively.

Another main limitation is the lack of specific guidelines for including the X-chromosome in association analysis. Although we discussed a number of practical strategies and approaches that can be followed for the analysis of X-chromosome, particularly in the context of GWAS and eQTL mapping, the application of the tests discussed are based on very specific scenarios. Currently, there is no consensus on a single best way for the analysis of X-chromosomal variants, and, in general, the selection for the association tests depends on several criteria. These criteria include but are not restricted to the underlying XCI model (escape, random or skewed), HWE, sex-specific alleles and whether adjustment for confounding variables is required (Figure 1). Generally, the approaches discussed above have corrected type 1 errors and have good statistical power [16, 17, 36] when the assumptions for each model are met (Figure 1). However, when the whole X-chromosome is scanned for associations, there may be extreme variations in the difference between male and female allele frequencies. In such an extreme case, regression-based association testing can be more appropriate and robust as it allows the integration of sex as a covariate [14, 36]. Overall, to decide which statistical model should be applied to our data, one strategy is to first estimate the degree of skewness related to XCI using tools, such as XCIR and GCTA, as discussed above. However, in practice, it is recommended to use a combination of association tests while scanning the full X-chromosome, since not all loci are subject to XCI. Lastly, when sex-specific variations in effect sizes or single-sex effects are expected, a sex-stratified approach can provide an increase in power to detect associations.

In addition to the particularities required for GWAS, we discussed strategies utilized by eQTL studies, which include X-chromosomal data. Although the statistical approaches used are straightforward, there are currently no standard guidelines for the inclusion of X-chromosome. In particular, eQTL analysis is often performed by the inclusion of sex as an additional covariate, which computationally adjusts for differences between males and females. Interestingly, these studies have shown a significant depletion of cis-eQTLs on the X-chromosome compared with autosomes. In contrast, increasing enrichment of sex-interacting eQTLs were observed on the X-chromosome, which may aid in elucidating underlying mechanisms of differential gene regulation between males and females [40, 41]. Therefore, these findings warrant more studies in the future.

Genetic associations to the X-chromosome in diseases are of special interest, including autoimmune and infectious diseases, given the sex bias observed in disease prevalence. A recently published review presented all suggestive associations on the X-chromosome (*P* <10$^{-5}$) to 17 different infectious diseases extracted from the GWAS catalog. Only 23 SNPs were associated at a genome-wide significant level with the following three infections: smallpox, influenza virus A subtype H1N1 and tuberculosis. Of note, only one out of 17 studies performed a sex-stratified analysis, while the rest combined males and females. One could speculate that this might be due to the loss of power while analyzing the two sexes separately. However, even biobanks with large sample sizes, such as the United Kingdom (UK) Biobank or the National-wide Network of Finnish Biobanks (FinnGen) analyze the X-chromosome combining males and females. In addition to infectious diseases, autoimmune diseases (ADs) are interesting case studies for investigating the role of X-chromosome in disease risk. Many ADs are sexually dimorphic in symptoms and differ significantly in prevalence between sexes, suggesting a potential contribution of X-chromosome in disease risk. A previously published study reanalyzed GWAS data from 16 autoimmune and related diseases and found several X-linked genes associated with a risk to ADs, such as Crohn’s disease [49]. Another example is systemic lupus erythematosus (SLE). Recent studies have identified several X-linked genes associated with SLE, such as *IRAK1*, *TLR7*, *MECP2* and *PRPS2* [50, 51, 52]. Most of these studies used case/control cohorts, consisting of females, and therefore have not mentioned sex-specific genetic effects.

Analysis of the X-chromosome is especially relevant in modern-day, when recent studies have shown evidence that males suffer from an increased risk of developing severe symptoms and thus are more likely to die from coronavirus disease 2019 (COVID- 19), caused by the severe acute respiratory syndrome coronavirus 2 (SARS-CoV-2) virus [53]. Recent studies have revealed several X-linked genes that play a role in COVID-19. One of these genes is angiotensin-converting enzyme 2 (*ACE2*), which is critical for the entry of SARS-CoV-2 into cells [54]. In addition, it was shown that *ACE2* escapes XCI in females, which may partially explain the sex bias in COVID-19 risk to severe disease [9, 38]. This demonstrates why more genetic studies should include the X-chromosome in their analysis. Closing this gap would greatly help in revealing the genetic basis of many complex diseases, especially those with a sex-bias [24, 42]. It is however important to note that while several diseases display a sex bias, this may be due to reasons other than genetic variations on the X-chromosomes, such as sex hormones [55].

## Conclusions and future perspectives

In this review, we highlighted a number of practical strategies and approaches that can be followed for the analysis of X- chromosome, particularly in the context of GWAS and eQTL mapping. However, there are still many limitations due to the complexity of this chromosome. Several tools and robust pipelines have been developed to facilitate the inclusion of X-chromosome in association studies. These tools allow the assessment of the skewness of XCI and the testing of different models of XCI to decide on which model best explains the variance of the phenotype of interest. Reanalysis of the X-chromosome data available from previous GWA studies using appropriate models followed up with integration with functional data can be a critical step toward understanding X-linked associations. Ultimately, this will shed light on the underlying biology and risk to diseases and traits that present a sex-bias.

**Key Points**

- Complex diseases/traits present sexual dimorphic prevalence which points toward a potential contribution of the X-chromosome.
- Quality control and imputation of X-chromosome genetic data require special attention to account for its unique properties.
- Selection of statistical tests to identify associations with X-chromosome loci depends on the underlying X-chromosomal inactivation (XCI) model, HWE, sex-specific alleles and confounding variables.

## Author contributions statement

N.K., V.M. and I.R wrote the manuscript and all authors revised the manuscript.
